# Molecular characterization of an MYB transcription factor from a succulent halophyte involved in stress tolerance

**DOI:** 10.1093/aobpla/plv054

**Published:** 2015-05-18

**Authors:** Pushp Sheel Shukla, Parinita Agarwal, Kapil Gupta, Pradeep K. Agarwal

**Affiliations:** 1 Wasteland Research Division, CSIR-Central Salt and Marine Chemicals Research Institute (CSIR-CSMCRI), Council of Scientific and Industrial Research (CSIR), Gijubhai Badheka Marg, Bhavnagar 364 002, Gujarat, India; 2 Academy of Scientific and Innovative Research, CSIR-Central Salt and Marine Chemicals Research Institute (CSIR-CSMCRI), Council of Scientific and Industrial Research (CSIR), Gijubhai Badheka Marg, Bhavnagar 364 002, Gujarat, India

**Keywords:** Abiotic stress, halophyte, R2R3-MYB, *Salicornia brachiata*, transcription factor, yeast

## Abstract

Abiotic stresses like drought, salinity and extreme temperature significantly affect crop productivity. Plants respond at molecular, cellular and physiological levels for management of stress tolerance. Functional and regulatory genes play a major role in controlling these abiotic stresses through an intricate network of transcriptional machinery. Transcription factors are potential tools for manipulating stress tolerance since they control a large number of downstream genes. In the present study, we have isolated *SbMYB44* from a succulent halophyte, *Salicornia brachiata* Roxb. *SbMYB44* with an open-reading frame of 810 bp encodes a protein of 269 amino acids, with an estimated molecular mass of 30.31 kDa and an isoelectric point of 6.29. The *in silico* analysis revealed that the SbMYB44 protein contains the conserved R2R3 imperfect repeats, two SANT domains and post-translational modification sites. The *SbMYB44* transcript showed up-regulation in response to salinity, desiccation, high temperature, and abscisic acid and salicylic acid treatments. The SbMYB44 recombinant protein showed binding to dehydration-responsive *cis*-elements (RD22 and MBS-1), suggesting its possible role in stress signalling. Overexpression of SbMYB44 enhanced the growth of yeast cells under both ionic and osmotic stresses.

## Abstract (precis)

Abiotic stresses cause dramatic changes in agricultural productivity. Plants encounter a wide range of biotic stresses and have evolved mechanisms to increase tolerance through both physical adaptations and interactive molecular and cellular changes that begin after the onset of stress. Transcription factors regulate the gene expression associated with both abiotic and biotic stresses, growth, metabolism, and plant development. *SbMYB44* is an R2R3 type transcription factor involved in stress regulation in an extreme halophyte, *Salicornia brachiata. SbMYB44* binds to the *cis* elements of a stress responsive promoter and confers stress tolerance. In future, SbMYb44 may prove to be a candidate gene for developing stress tolerance in crop plants.

## Introduction

Evolution has equipped plants with a wide spectrum of adaptations to combat environmental perturbations (Dinakar and Bartels 2013). Abiotic stresses, like drought, salinity and extreme temperature, negatively influence plant growth and yield. Plants respond to these stresses by changes in both regulator and functional genes. Transcriptional control is the major mechanism that regulates gene expression associated with both abiotic and biotic stresses, growth, metabolism and development of plants, through an intricate network of transcription factors (TFs). The transcriptional machinery has evolved from primitive to complex life forms with more regulator genes in plants when compared with yeast (Riechmann *et al*. 2000). Transcription factors can be classified into more than 50 different families on the basis of their DNA-binding domains (Riechmann *et al*. 2000). These regions are responsible for binding to the specific *cis-*elements in the promoters. The control and co-ordination among specific set of genes is accomplished by the combined interaction among TFs, between TFs and non-DNA-binding proteins, and between TFs and *cis*-regulatory elements (Agarwal *et al*. 2013). The TFs correspond to 5–7 % of the total genes and have increased in number, presumably due to the complexity of plant metabolism (Shiu *et al*. 2005).

MYB proteins constitute one of the largest TF families in plants (Lindemose *et al*. 2013). These proteins possess the highly conserved MYB domains comprising single, double or triple imperfect repeats. Each repeat contains 50–53 amino acids (aa) with three α-helices. The second and third helices of each repeat have three regularly spaced tryptophan (W) residues forming a helix–turn–helix (HTH) with a hydrophobic core (Ogata *et al*. 1996). The third helix of each repeat serves as ‘recognition helix’ facilitating intercalation of DNA in the major groove (Jia *et al*. 2004). MYB proteins are classified into four subfamilies namely R1-MYB, R2R3-MYB, 3R-MYB and 4R-MYB depending on the number of adjacent repeats in the MYB domain (Dubos *et al*. 2010). The first plant MYB, c-MYB-like TF, involved in anthocyanin biosynthesis, was isolated from maize (Paz-Ares *et al*. 1987); since then a number of MYB TFs have been isolated and characterized from different plants. The R2R3-MYB TFs are highly represented in plants with 157 in maize (Du *et al*. 2012) and more than 100 in *Arabidopsis* (Yanhui *et al*. 2006). The R2R3-MYBs are considered to have evolved from an R1R2R3-type ancestral gene, from which the first repeat was lost (Braun and Grotewold 1999). The three- and four-repeat MYB TFs have also been identified in *Arabidopsis* (Braun and Grotewold 1999). The role of different members of the MYB family have been studied during cellular processes including growth and development (*AtMYB123*, Nesi *et al*. 2000), embryogenesis (*AtMYB118*, Zhang *et al*. 2009), abiotic stresses (*OsMYB3R*-2, Dai *et al*. 2007; *OsMYB55*, El-Kereamy *et al*. 2012; *CaMYB*, Seong *et al*. 2008; *TaPIMP1*, Zhang *et al*. 2012; *TaMYB73*, He *et al*. 2012; *OsMYB*, Yang *et al*. 2012), defence responses to pathogens (*BOS-1*, Mengiste *et al*. 2003; *TiMYB2R*-1, Liu *et al*. 2013) and as repressors and enhancers of lignification (*EgMYB1*, Legay *et al*. 2010; *AtMYB61*, Newman *et al*. 2004). MYBs are involved in the abscisic acid (ABA)-, salicylic acid (SA)- and jasmonic acid (JA)-dependent signalling pathways (Abe *et al*. 2003; Shan *et al*. 2009).

Although MYB TFs have been isolated from a large number of glycophytes, their characterization from halophytes is limited. *Salicornia brachiata* Roxb. (Amaranthaceae) is a leaf-less, annual, succulent, obligate halophyte growing abundantly in the coastal area of Gujarat, India. *Salicornia brachiata* accumulates salt in its stems and can survive as high as 2 M NaCl in the field (Reddy *et al*. 1992, 1993). *Salicornia brachiata* accumulates NaCl to 30–40 % of its dry weight. Therefore, its biomass was utilized successfully at our institute (CSIR-CSMCRI) for the preparation of nutrient-rich salt of plant origin (US patent no. 6 929 809). *Salicornia brachiata* adapts to high salinity and drought by accumulation of compatible osmolytes and reducing stress-induced oxidative damage (Parida and Jha 2010, 2013). Identification of stress-induced ESTs in *Salicornia* showed that 4.8 % ESTs belonged to stress-tolerant gene category (Jha *et al*. 2009). SbDREB2a, a DREB TF isolated from *S. brachiata*, induced tolerance to both salinity and desiccation in transgenic tobacco (Gupta *et al*. 2010, 2014). In the present study, we report an SbMYB44, an R2R3-type MYB TF from *Salicornia* and its transcript regulation in response to different developmental stages, abiotic stresses and by signalling molecules. The SbMYB44 also showed binding with different *cis*-elements of abiotic stress-related promoters. Overexpression of SbMYB44 enhanced the growth of yeast cells in the presence of salinity and dehydration stress.

## Methods

### Plant material and stress treatment

Seeds of *S. brachiata* were harvested from dried plants collected from the coastal area near Bhavnagar, Gujarat, India (GPS 21°35.634′N and 72°16.786′E). The seeds were germinated and grown in plastic pots with garden soil under natural conditions. One-month-old seedlings were transferred to the hydroponic medium (1/2 major and minor salts of Murashige and Skoog's medium, Murashige and Skoog 1962) in a plant growth chamber (CU-36L, Percival Scientific, Perry, IA, USA) with light/dark (300–350 µmol m^−2^ s^−1^ spectral flux photon of photosynthetically active radiations) cycle of 16/8 h at 25 °C. After acclimatization, plants were treated with 250 mM NaCl, desiccation (wrapped in tissue paper at room temperature), 100 µM ABA, 2.5 mM SA, and heat (37 °C) for 0, 0.5, 1, 6, 12, 24 and 48 h. The treated plant samples were frozen in liquid nitrogen and stored at −80 °C for transcript analysis.

### Isolation of *SbMYB44* gene

Total RNA was isolated from 1-month-old seedlings treated with 500 mM NaCl for 15 days by the guanidinium thiocyanate method (Chomczynski and Sacchi 1987). The total RNA (2.5 µg) was used for first-strand cDNA synthesis using RevertAid cDNA synthesis kit (Thermo Scientific). Degenerate primers (forward 5′-AYGGAYCGGRTYAARGGYCCRTGGAG-3′ and reverse 5′-TCTTTATCATYYCYTGCATCAC-3′) were designed from the conserved region of nucleotide alignment made from MYB sequences of *Capsicum annuum* (EF222025), *Malus domestica* (DQ074461), *Vitis vinifera* (AY953543), *Glycine max* (DQ822911) and *Solanum tuberosum* (AF122051). A polymerase chain reaction (PCR) was carried out using cDNA as template with 150 ng of primers, 200 µM dNTPs and 2.5 U *Taq* DNA polymerase in a 50 µL reaction volume under the following conditions: 94 °C, 5 min for 1 cycle; 94 °C, 1 min; 55 °C, 1 min and 72 °C, 1 min for 35 cycles; and last 72 °C, 7 min for 1 cycle. The amplicon was gel-purified, cloned in pJET 1.2 vector (Thermo Scientific) and sequenced at Macrogen (Seoul, Korea). After confirmation of the sequence by BLAST search, the 5′ and 3′ rapid amplification of cDNA ends (RACE) were conducted. The 5′ RACE was done using Invitrogen kit (USA) with the help of the following gene-specific primers: GSP R1 (5′-GATCTCCGAGAATTTCCTCTTC-3′), GSP R2 (5′-ACCGAACCTAGCGTGAGCTTTGA-3′) and GSP R3 (5′-GGCACGATGTTCAACTTCCGGTG-3′). For 3′ RACE, cDNA was synthesized using the PK1 primer (5′-CCAGTGAGCAGAGTGACGAGGACTCGAGCTCAAGC(T)_17_-3′). The primary 3′ RACE PCR was carried out using an adaptor primer PK2 (5′-CCAGTGAGCAGAGTGACG-3′) and a GSP1-F primer (5′-CGCTAGGTTCGGTAACAAATGG-3′). The secondary PCR was carried out using 1 : 50 dilution of primary PCR product with PK3 (5′-GAGGACTCGAGCTCAAGC-3′) and GSP2-F (5′-GTCTGATGTCAGCGTCTCTGG-3′) primers, and the amplified product was cloned in pJET 1.2 vector (Thermo Scientific) and sequenced. The sequence generated using degenerate primer amplification, 5′ RACE and 3′ RACE, was combined to obtain a full-length SbMYB44 sequence *in silico*. Furthermore, the full-length gene was amplified and cloned using SbMYB44 F (5′-ATGGAAAGCCACCAAACTTTATCATCG-3′) and SbMYB44 R (5′-TTAATGGAAATGACCGTACCGAGAAAGGGAT-3′) primers. For studying the genomic organization, the genomic DNA was PCR amplified with gene-specific primers SbMYB44 F and SbMYB44 R.

### *In silico* analysis

A phylogenetic tree of SbMYB44 was constructed with amino acid sequences of different MYB TFs using the maximum-likelihood method by the MEGA 6 software.

The secondary structure of SbMYB44 was predicted using the PSIPRED software. Solvent accessibility of a protein is determined using Predict protein server (Jones 1999; Rost *et al*. 2004). MEMSAT3 and MEMSATSVM software were used to predict the transmembrane helix of SbMYB44 (Nugent and Jones 2009). Conserved domains in SbMYB44 were identified via the SMART software (http://smart.embl-heidelberg.de/). A nuclear localization signal of SbMYB44 was predicted using cNLS mapper (http://nls-mapper.iab.keio.ac.jp/cgi-bin/NLS_Mapper_form.cgi). Primary SbMYB44 and other MYB protein sequences were analysed for N-glycosylation sites, protein kinases sites, myristoylation sites and phosphorylation sites by Motif Scan (http://myhits.isb-sib.ch/cgi-bin/motif_scan). Sequence logos for R2 and R3 MYB repeats of SbMYB44 were obtained from http://weblogo.berkeley.edu/logo.cgi (Crooks *et al*. 2004).

### Expression analysis of *SbMYB44* transcript by real-time PCR

Total RNA was extracted from various stress-treated *Salicornia* shoots and first-strand cDNA was synthesized using RevertAid cDNA synthesis kit (ThermoScientific). Real-time PCR was performed in a CFX detection system (Bio-Rad, USA) with 1× Sso Advanced SYBR green supermix (Bio-Rad) using 60 ng of SbMYB44 primers (forward 5′-CTGACGTTGAGTTTCATCGCCCT-3′ and reverse 5′-GAGGAGGTGAATCGGAAGAAA-3′) and β-tubulin primers (forward 5′-GGAGTCACCGAGGCAGAG-3′ and reverse 5′-ATCACATATCAGAAACCACAAAG-3′) under the following PCR conditions: 94 °C, 2 min for 1 cycle; 94 °C, 30 s, 55 °C, 30 s and 72 °C, 30 s for 40 cycles; 72 °C, 7 min for 1 cycle. At the end of the PCR cycles, the products were subjected to melt curve analysis to verify the specificity of PCR amplification. Three independent experiments were performed and the relative gene expression was determined using the Livak method (Livak and Schmittgen 2001). Transcript expression of *SbMYB44* at 0 h for each treatment served as control and was used for normalization of *C*_T_ values of each treatment (Livak and Schmittgen 2001).

### Transactivation assay of SbMYB44

A yeast one-hybrid assay was performed to study the transcriptional activation of the SbMYB44 protein (He *et al*. 2012). *SBMYB44* cDNA was amplified using SbMYB44 F (5′-CCGGAATTCATGGAAAGCCACCAAACTTTA-3′) and SbMYB44 R (5′-CGCGGATCCTTAATGGAAATGACCGTACCG-3′) with the EcoR1 and BamH1 flanking restriction sites, respectively. The digested *SbMYB44* was cloned in EcoRI and BamHI sites of pGBKT7 vector (Clontech). Plasmids of *SbMYB44* and vector alone were transformed separately into yeast strain AH109 (Clontech) and grown on synthetic dropout medium lacking tryptophan (SD/-Trp). *HIS3* activity was assessed by conducting a viability test on a histidine-lacking medium (SD/-His). *LacZ* activity was tested by performing the galactosidase filter lift assay (Ma *et al*. 2009).

### Cloning of *SbMYB44* cDNA in pET28a expression vector and purification of recombinant protein

*SbMYB44* open-reading frame (ORF) was PCR amplified using SbMYB44 PF (5′-CCGGAATTCATGGAAAGCCACCAAACTTTA-3′) and PR (5′-CCGCTCGAGTTAATGGAAATGACCGTACCG-3′) with flanking restriction sites of EcoRI and XhoI sites, respectively, and cloned in pJET1.2 vector. The digested *SbMYB44* was cloned in pET28a vector. Plasmids of pET28a-*SbMYB44* and vector alone were transformed separately in *Escherichia coli* BL-21 Star (DE3) cells (Invitrogen). Recombinant protein was induced with 1 mM isopropyl β-d-1-thiogalactopyranoside (IPTG) at 37 °C for 2, 4, 6 h and purified under native conditions using Ni–NTA Fast Start Kit, using manufacturer's protocol (Qiagen, Germany).

### DNA probes and gel mobility shift assay

Complementary oligonucleotides of *RD22* (F 5′-CTTCTAACCACTACGTGCCTTCTGCTCCTTCTGC-3′, and R 5′-GCAGAAGGAGCAGAAGGCACGTAGTGGTTAGAAG-3′ (Abe *et al*. 1997) and MBS*-1* (F 5′-CGAGACACCCTAACTGACACACATTCT-3′ and R 5′-AGAATGTGTGTCAGTTAGGGTGTCTCG-3′) (Nakagoshi *et al*. 1990) were synthesized having unique binding sites (underlined). The dehydration-responsive element (DRE) from *Arabidopsis RD29a* promoter (F 5′-TAAAAG ATATACTACCGACATGAGTTC CAA AAAGC-3′ and R 5′-GCTTTTTGG AACTCATGTCGGTAGTATATCTTT TA-3′) was used as a negative control (Liu *et al*. 1998; Agarwal *et al*. 2007). Complementary oligonucleotides (2 µg) from different promoter elements were annealed separately using annealing buffer (100 mM Tris–HCl, 5 mM NaCl, 10 mM ethylenediaminetetraacetic acid (EDTA)) by incubating at 60 °C for 5 min and then at 37 °C for 15 min. The electrophoretic mobility shift assay (EMSA) was carried out using 80 ng of annealed probe with 40 µg of purified recombinant SbMYB44 protein in binding buffer [15 mM HEPES, 35 mM KCl, 4 mM EDTA (pH 8.0), 1 mM DTT, 1 mM MgCl_2_ and 6 % glycerol] at room temperature for 20 min. The reaction was terminated by adding 10× DNA loading dye and fractionated on non-denaturing 10 % acrylamide gel in 0.5× tris-borate-EDTA buffer. The gel was stained with SYBR Green dye.

### Stress tolerance assay of *Saccharomyces cerevisiae* under different stresses

The *SbMYB44* ORF was cloned in the EcoRI and XhoI sites of the yeast expression vector pYES2 (Invitrogen). The plasmids of pYES2-*SbMYB44* and vector alone were transformed separately in yeast strain W303 using Yeastmaker yeast transformation system (Clontech). A stress tolerance assay of recombinant yeast cells was performed as described by Li *et al*. (2014) with minor modifications. Yeast cells having pYES2-*SbMYB44* and vector alone were grown in SD/-Ura broth for 24 h at 30 °C. After adjusting the OD_600_ to 0.4, 500 µL of culture was added to 10 mL of induction medium (SD/-Ura broth supplemented with 2 % galactose) and grown for 36 h to promote the expression of *SbMYB44* gene. The cultures were diluted to an OD_600_ 0.6, and 500 µL of culture was inoculated in 10 mL SD/-Ura containing 2.5 M NaCl, 5.0 M NaCl, 15 % polyethylene glycol (PEG 6000) equivalent to −0.295 MPa of osmotic potential and 30 % PEG 6000 equivalent to −1.027 MPa of osmotic potential and incubated at 30 °C for 36 h. After stress treatment, the cultures were serially diluted (10^0^, 10^−1^, 10^−2^, 10^−3^, 10^−4^) and 7 µL from each dilution were spotted on SD/-Ura medium and incubated at 30 °C for 3 days.

### Statistical analysis

Each experiment was performed three times and data were recorded. One-way analysis of variance with replicates was performed in Microsoft Excel. Critical difference values were calculated at *P* ≤ 0.05 to determine the significance of difference between the means, across the treatments, of transcript expression at various intervals. The mean values that were significantly different within treatment from each other are indicated by different letters. The standard deviation was calculated to show the variation in the replicates.

## Results

### Cloning and sequence analysis of *SbMYB44*

**Figure 1.**
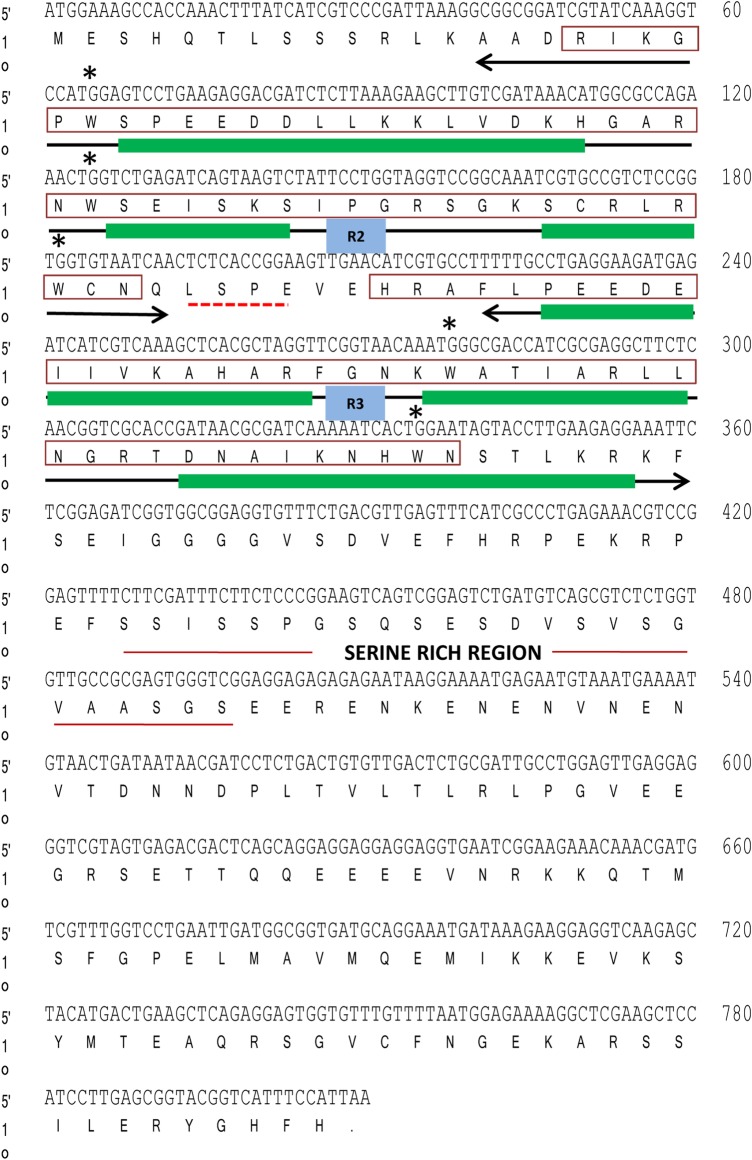
Schematic representation of the SbMYB44 TF sequence. R2 and R3 repeat DNA-binding domains are underlined with double-headed arrows. Conserved tryptophan residues (W) are marked by asterisks and green bars represent α-helices in R2R3 repeats. SANT domains are demarcated by red outlined box. LSPE motif is marked with red-dashed line. Serine rich region is represented with red solid line.

**Figure 2.**
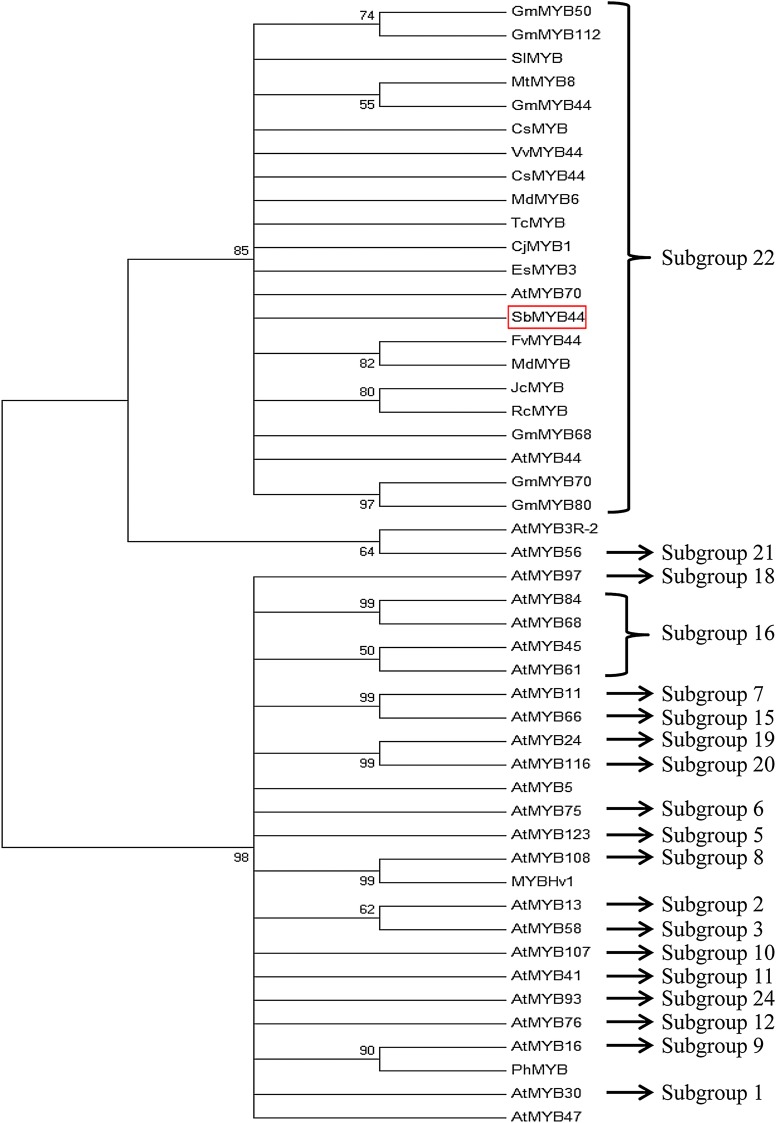
The phylogenetic relationship of SbMYB44 was constructed using the maximum-likelihood method. The protein sequences used for the construction of phylogenetic tree are as follows: *Salicornia brachiata*, SbMYB44 (AHN50422), *Citrus sinensis*, CsMYB (ABQ10816), *Cucumis sativus*, CsMYB44 (XP004141899), *Fragaria vesca*, FvMYB44 (XP004305954), *Glycine max*, GmMYB50 (NP001238087), GmMYB68 (NP001235715), GmMYB70 (NP001237693), GmMYB80 (NP001237709), GmMYB112 (NP001235142), GmMYB44 (XP003524661), *Malus domesticus*, MdMYB (ADL36760), MdMYB6 (AAZ20429), *Medicago truncatula*, MtMYB8 (ABR28330), *Solanum lycopersicum*, SlMYB (XP004238123), *Theobroma cacao*, TcMYB (XP007047395), *Vitis vinifera* VvMYB44 (XP002285015), *Arabidopsis thaliana* AtMYB44 (AED98326), AtMYB3R-2 (AF151647), AtMYB56 (AF062891), AtMYB45 (AF062883), AtMYB61 (AF62896), AtMYB97 (AF176004), AtMYB84 (Y14209), AtMYB30 (AF062873), AtMYB47 (AF062885), AtMYB13 (Z50869), AtMYB58 (AF062893), AtMYB24 (AF175987), AtMYB116 (AF334815), AtMYB11 (AF062863), AtMYB66 (AF062900), AtMYB5 (X90380), AtMYB123 (AF371981), AtMYB76 (AF175992), AtMYB41 (AF062882), AtMYB107 (AF249310), AtMYB93 (AF062917), AtMYB16 (X99809), *Hordeum vulgare*, MYBHv1 (CAA50224).

An amplicon of 630 bp was amplified using degenerate primers. After confirming its sequence by NCBI BLAST, 5′ and 3′ RACE were conducted to obtain the full-length cDNA. The 5′ and 3′ RACE showed amplification of 364 and 533 bp, respectively. The SbMYB44 cDNA comprised an ORF of 810 bp encoding an R2R3-type MYB protein of 269 aa with a calculated molecular weight of 30.31 kDa and an isoelectric point of 6.29 (GenBank accession number: KJ027517). The amino (N)-terminal region of SbMYB44 contain the conserved R2R3 imperfect repeats, which are involved in binding to target DNA sequences **[see Supporting Information—Fig. S1]**. The R2 and R3 repeats showed the presence of three and two Trp (W) residues, respectively. SbMYB44 contains two SANT domains, one present in the R2 repeat (18–63 aa) and the other present in the R3 repeat (71–113 aa). The LSPE motif is present in the linker region of R2 and R3 repeats (Fig. 1). The PSIPRED protein structure prediction server showed the presence of 33.46 % helix and 66.54 % loop **[see Supporting Information—Fig. S2A and B]**. Solvent accessibility of the protein revealed that the 46.47 % region is accessible to solvents, the 17 % region to intermediate exposure while rest of 35.69 % is buried [inaccessible to solvents, **Supporting Information—Fig. S2C**]. The region between 86 and 101aa showed transmembrane helix having an extracellular carboxy (C)-terminal region and a cytoplasmic N-terminal region while the region between 191 and 200aa had an extracellular N-terminal region and a cytoplasmic C-terminal region **[see Supporting Information—Fig. S3]**. The PROSITE analysis revealed three glycosylation motifs (41–44, 113–116, 180–183 aa), two cAMP- and cGMP-dependent protein kinase phosphorylation sites (118–121, 216–219 aa), six casein kinase II phosphorylation sites (23–26, 129–132, 150–153, 164–167, 206–209, 260–263 aa), five protein kinase C phoshphorylation sites (9–11, 53–55, 56–58, 115–117, 192–194 aa) and three myristoylation sites (160–165, 197–202, 249–254 aa). The maximum-likelihood phylogenetic tree of SbMYB44 was constructed on the basis of alignment of the complete deduced polypeptide sequences of different MYB TFs. Bootstrap values from 1000 replicates were used to assess the robustness of the tree. In the phylogenetic tree, bootstrap values >50 are shown. The phylogenetic analysis revealed that SbMYB44 is clustered in subgroup 22 of the MYB family with close homology to FvMYB44 and MdMYB from *Fragaria vesca* and *Malus domestica* (Fig. 2).

### Transcript expression of *SbMYB44*

**Figure 3.**
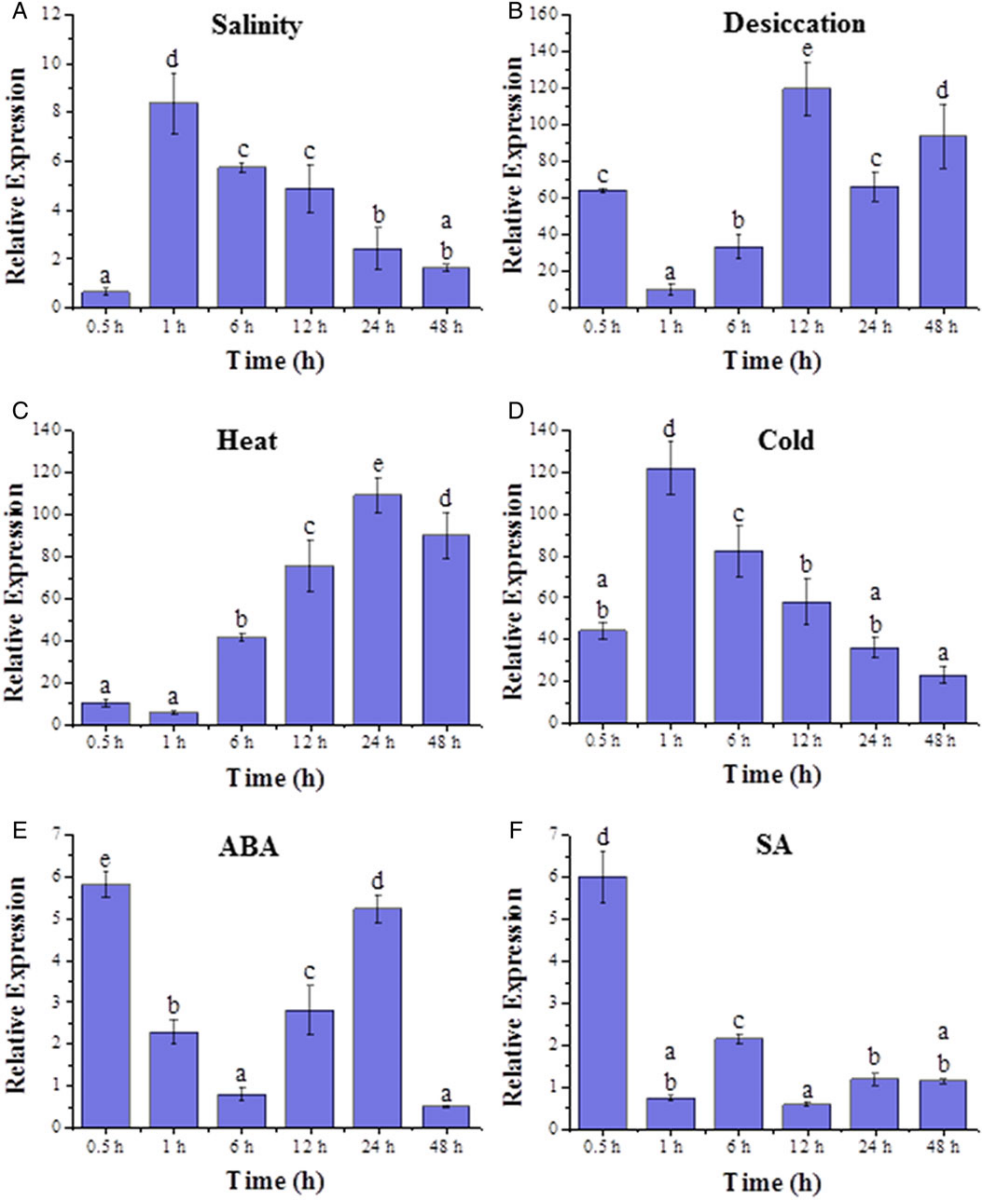
Transcript profiling of *SbMYB44* with different stress conditions: (A) NaCl, (B) desiccation, (C) heat, (D) cold, (E) ABA and (F) SA.

*Salicornia brachiata* is adapted to different stress conditions such as salinity and drought, and to examine whether SbMYB44 contributes to its adaptability, transcript expression of *SbMYB44* was studied in the presence of different stresses and stress-related phyto-hormones. The results showed that *SbMYB44* expression was induced significantly by salt, desiccation, cold, high temperature, ABA and SA. In the presence of salinity, the maximum transcript accumulation of *SbMYB44* was observed at 1 h (8-fold) and then reduced with increasing time (Fig. 3A). Under desiccation stress, *SbMYB44* showed an early induction at 0.5 h (64-fold) and reaches its maximum level (120-fold) at 12 h (Fig. 3B). In response to high temperature, transcript levels showed their highest expression at 24 h of exposure (118-fold) (Fig. 3C). During cold treatment, the expression was found maximum at 1 h (120-fold) and then reduced (Fig. 3D). In the presence of ABA, *SbMYB44* was maximally expressed at 30 min (6-fold), decreased till 6 h and again increased at 12 and 24 h (Fig. 3E). In the presence of SA, the expression was up-regulated at 0.5 h (6-fold) and then gets down-regulated (Fig. 3F).

### Transcriptional activation of SbMYB44

**Figure 4.**
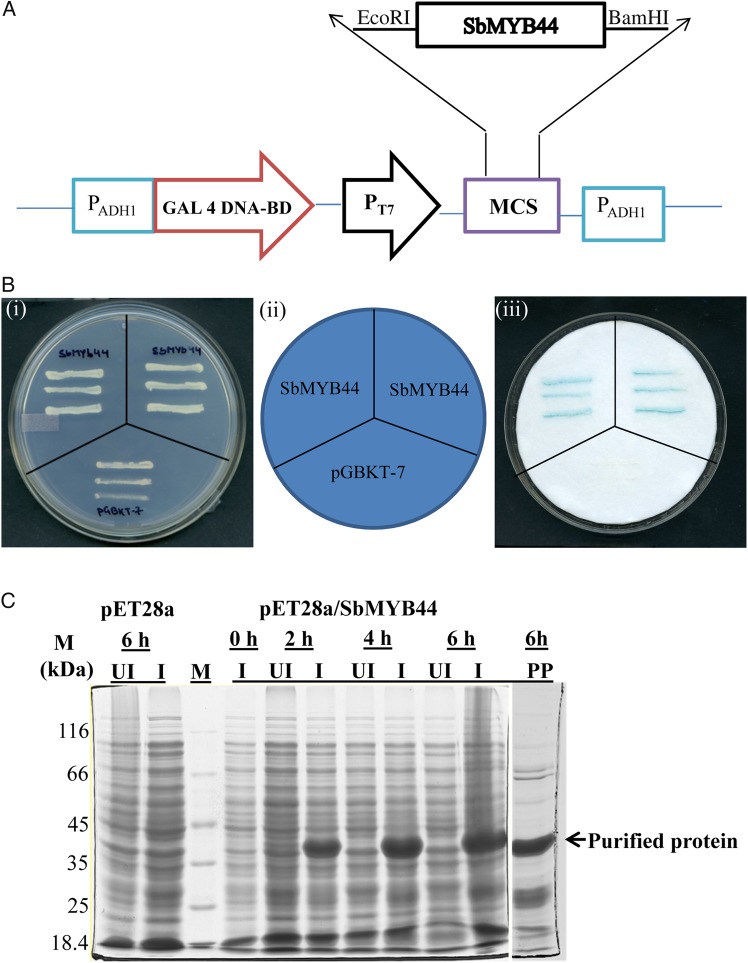
Transactivation assay of SbMYB44. (A) Full-length *SbMYB44* ORF cloned in pGBKT7 vector. (B)(i) Transformed yeast cell (AH109) containing pGBKT7 + *SbMYB44* and pGBKT7 alone grown on SD/-Trp/-His/-Ura medium. (ii) Schematic representation of the plating. (iii) Yeast cells transferred on filter paper showed β-galactosidase (encoded by LacZ) activity using X-gal staining. (C) SDS–PAGE analysis of expression of recombinant SbMYB44 protein in *E. coli* BL-21 Star (DE3) cells. M, marker; UI, un-induced protein; I, induced protein.

A yeast GAL4 system was used to investigate the transcriptional activation of SbMYB44. The GAL4 DNA-binding domain-*SbMYB44* recombinant plasmid was transformed into yeast strain AH109 and assayed for its ability to activate transcription of the dual reporter gene *His3* and *LacZ*. Yeast cells with recombinant plasmid harbouring *SbMYB44* grew on SD/-His medium, and showed blue colour in X-gal solution (Fig. 4A and B) indicating its activity as a TF.

### Binding of SbMYB44 recombinant protein to *cis*-elements

The 6× His-Tag SbMYB44 recombinant protein of 30.31 kDa was induced with 1 mM IPTG for different time periods. The protein showed a maximum induction at 6 h; therefore, the protein was induced for 6 h and purified to near homogeneity (Fig. 4C).

**Figure 5.**
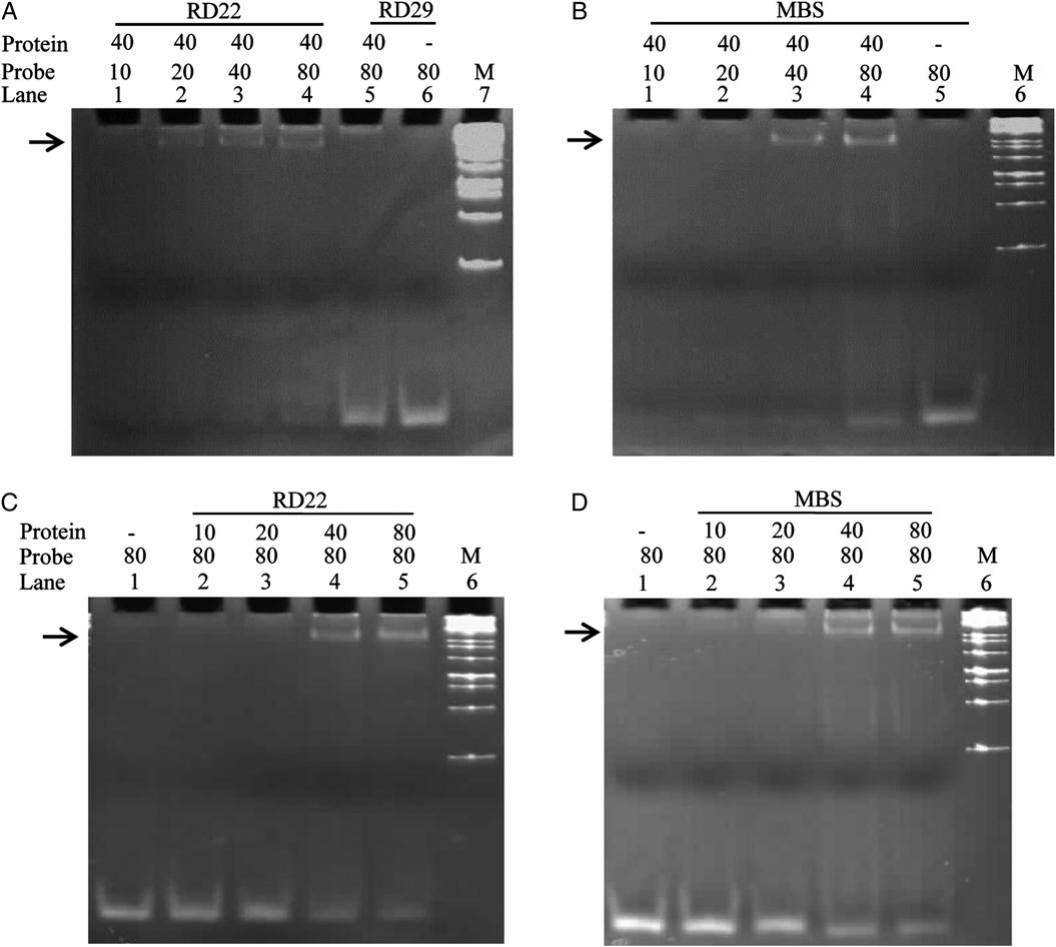
The EMSA study showing binding of SbMYB44 protein with different *cis-*elements. (A and B) Binding of 40 ng of protein with different amounts of probe. (C and D) Binding of 80 ng of probe with different amounts of protein.

The EMSA was performed to study the involvement of SbMYB44 in different signalling pathways during plant development and stress by interaction with *cis*-elements of different promoters like *RD22* (dehydration responsive) and *MBS-1* (dehydration responsive) (Fig. 5). The *RD29 cis*-element was used as a negative control and it did not show binding with a recombinant protein. The SbMYB44 protein showed increased binding with increasing concentration of the probe, the maximum binding was observed using 80 ng probe with all the *cis*-elements (Fig. 5A and B). Different amounts of recombinant protein were also analysed for binding reaction with 80 ng probes (Fig. 5C and D). The maximum binding was found with 40 and 80 ng proteins.

### *SbMYB44* confers enhanced stress tolerance in yeast

**Figure 6.**
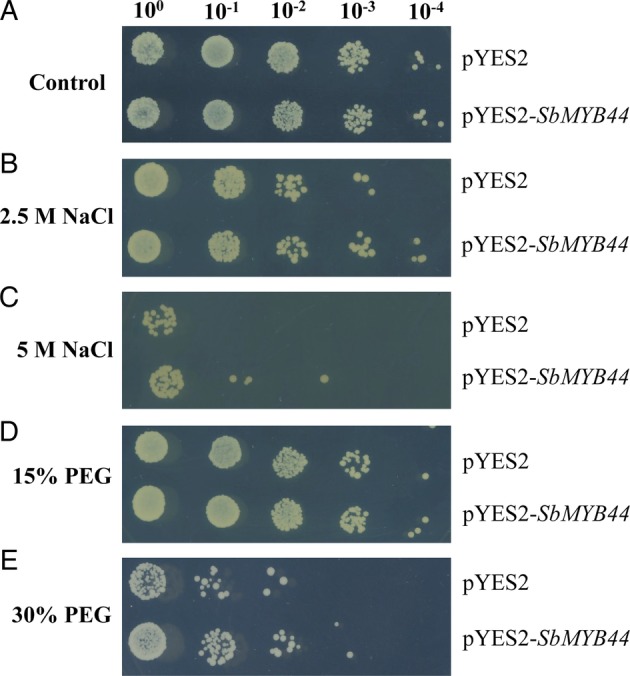
Spot assay of yeast (W303) cells with pYES2 and pYES2-SbMYB44 on SD/-Ura basal medium after stress treatment. (A) Control, (B and C) 2.5 and 5 M NaCl and (D and E) 15 and 30 % PEG.

In the presence of different stresses, *SbMYB44* ORF, driven by a galactose-inducible promoter, showed better growth in recombinant *S. cerevisiae* (W303) compared with wild-type cells. The growth rate of pYES2-*SbMYB44*-transformed yeast cells was similar to the growth rate of pYES2-transformed yeast cells under non-stress conditions (Fig. 6A). In the presence of 2.5 and 5.0 M NaCl, pYES2-*SbMYB44-*transformed cells showed higher growth than pYES2 alone (Fig. 6B and C). Similarly, pYES2-SbMYB44-transformed yeast cells produce more cells than pYES2 alone, in the presence of the dehydration stress with 15 and 30 % PEG (Fig. 6D and E). These results indicate that SbMYB44 was functional in yeast and improved its tolerance to salinity and dehydration stress.

## Discussion

Plant TFs play important roles in regulating the gene expression during development and abiotic stress tolerance. MYB TFs form a large family of proteins that are involved in an array of functions such as primary and secondary metabolism, regulation of plant development, regulation of cell fate and identity and in response to biotic and abiotic stress tolerance (Dubos *et al*. 2010; Agarwal *et al*. 2013). In this study, an R2R3-type *SbMYB44* was isolated from *S. brachiata*. The *in silico* analysis revealed that SbMYB44 has similar HTH repeats as present in other R2R3-type MYBs (Romero *et al*. 1998). SbMYB44 possesses a bipartite type of nuclear localization signal similar to MdMYB, FvMYB44 and GmMYB44. The motif scan comparison of SbMYB44 showed the presence of myristoylation, glycosylation and other post-translational modification. These post-translational modifications could help to mediate the fine tuning of SbMYB44 activity, although no function has yet been known for these post-translational modifications. Myristoylation plays a vital role in membrane targeting and signal transduction in responses to environmental stress (Podell and Gribskov 2004); therefore, the presence of a myristoylation site suggests that it may play a role in regulating the activity of SbMYB44 in response to different stresses. The MYB DNA-binding domain of SbMYB44 possesses highly conserved and evenly distributed W residues, which are important for forming the hydrophobic core and maintaining three dimensional structure of the MYB repeats (Stracke *et al*. 2001). Similar to the R2R3-MYBs of maize and *Arabidopsis* (Dubos *et al*. 2010; Du *et al*. 2012), SbMYB44 also showed high conservation of phenylalanine residue, which replaces the first tryptophan in the R3 repeat. R2R3-MYB belonging to subgroup 22 have consensus sequence (GxFMxVVQEMIxxEVRSYM) but SbMYB44 had GPELMAVMQEMIKKEVKSYM at C-terminal end. Phylogenetic relationships revealed that SbMYB44 clustered with the member of subgroup 22, such as AtMYB44 and AtMYB70 (Dubos *et al*. 2010), and show close homology with MdMYB (53 %), FvMYB44 (58 %), GmMYB68 (57 %) and other members of the MYB family belonging to the subgroup 22. The C-terminal characteristics of SbMYB44 are consistent with its classification in R2R3-MYB subgroup 22 and may also suggest functional conservation between SbMYB44 and other subgroup members.

*SbMYB44* was induced by NaCl, desiccation, heat- and stress-related phyto-hormones (ABA and SA). The protein also showed binding with the drought-inducible promoter *RD22* and *MBS-1*. Similarly, Urao *et al*. (1993) showed transcript accumulation of *AtMYB2* in response to dehydration, salt, ABA and binding to the *cis*-element of *RD22* and *MBS-1* promoter (Urao *et al*. 1993; Abe *et al*. 2003). The *AtMYB44* showed strong induction with salt, drought, cold and ABA (Kranz *et al*. 1998), and stress tolerance by ABA-dependent stomatal closure in transgenic *Arabidopsis* (Jung *et al*. 2008). AtMYB20 is also highly expressed in the presence of NaCl, drought and ABA (Cui *et al*. 2013). Similarly, the expression of PtsrMYB, an R2R3-MYB from *Poncirus trifoliata*, is induced by different stresses (Sun *et al*. 2014). The expression of *SbMYB44* was also induced by the salinity, but the relative-fold induction was lower when compared with other stresses like heat, desiccation and cold. In the coastal region, the sea water is inundated twice in a month and remaining times the soil is almost dry and temperature is high, therefore, in addition to the salinity, *S. brachiata* also shows tolerance towards dehydration and temperature alteration. SbMYB44 might be a TF which is involved in tolerance of these plants to desiccation and temperature stress rather than salt and helps *S. brachiata* in relieving physiological drought condition. *SbMYB44* was induced both with ABA and SA, suggesting the involvement of ABA and SA in MYB regulation. A wheat MYB TaPIMP1 showed resistance against *Bipolaris sorokiniana* and to drought stress through activation of SA- and ABA-responsive downstream genes suggesting its role in cross-talk between abiotic and biotic stress signalling (Zhang *et al*. 2012).

MYB proteins have a highly conserved MYB DNA-binding domain across the eukaryotes including yeast, plants and animals (Lipsick 1996). The R2R3-type MYB TFs have evolved from MYB3R protein, and are closely related to vertebrate c-MYB and MYB TFs from other eukaryotic group such as slime moulds and ciliates (Feller *et al*. 2011); therefore, these proteins possibly existed before the divergence of plants and animals (Kranz *et al*. 2000). The most distant relative of c-MYB is the BAS1 gene of *S. cerevisiae*, involved in the regulation of HIS4 gene. Sequence analysis revealed that BAS1 binds to the PyAACG/TG (closely related MYB response element) (Luscher and Eisenman 1990). In our study, the SbMYB44 protein showed binding with different promoters having similar core sequence (TAACTG) motifs. Therefore, it might be possible that SbMYB44 protein confers stress tolerance in the yeast by binding to the similar core sequence.

We conclude that *SbMYB44* is an important gene for stress tolerance. It is an R2R3-type MYB TF that showed enhanced transcript accumulation under different stress condition. The SbMYB44 recombinant protein showed binding with different *cis*-elements of stress-related promoters like *RD22* and *MBSI* having similar core sequences, plausibly up-regulating the downstream genes under their control for stress tolerance. The enhanced tolerance observed in eukaryotic yeast highlights that SbMYB44 might be a potential TF for improved growth during ionic and osmotic stresses, and could be further used for developing stress-tolerant crop plants.

## Sources of Funding

The study was supported by Department of Science and Technology (DST) and Council of Scientific and Industrial Research (CSIR), New Delhi, India.

## Contributions by the Authors

K.G. and P.S.S. carried out gene cloning, and P.S.S. and P.A. carried out experiments for functional validation. P.K.A. and P.A. coordinated the experiments and finalized the manuscript. All authors read and approved the final manuscript.

## Conflict of Interest Statement

None declared.

## Supporting Information

The following additional information is available in the online version of this article –

**Figure S1.** Sequence logos for the (A) R2 and (B) R3 repeats of SbMYB44 MYB proteins. The overall height of each stack indicates the conservation of the sequence at particular position; whereas, the height of letters within each stack represents the relative frequency of the corresponding amino acid. The triangle indicates the positions of the conserved amino acid that is identical in other MYB proteins.

**Figure S2.** (A) Secondary structure prediction of SbMYB44 protein using the PSIPRED software, (B) secondary structure of SbMYB44 showing 33.46 % α helices and 66.54 % loop, (C) solvent accessibility of protein is determined using Predict protein server.

**Figure S3.** (A) Schematic diagram of the MEMSAT3 and MEMSATSVM prediction of SbMYB44 showing the presence of transmembrane helix (marked by grey region), (B) the region between amino acids 86 and 101 showing transmembrane helix having extracellular C-terminal and cytoplasmic N-terminal regions, while the region between 191 and 200 shows a transmembrane helix having extracellular N-terminal and cytoplasmic C-terminal regions.
